# Carbohydrate Counting: A Bibliometric Analysis with a Focus on Research

**DOI:** 10.3390/nu16193249

**Published:** 2024-09-26

**Authors:** Simge Yilmaz Kavcar, Gizem Köse, Kezban Esen Karaca Çelik, Aslı Çelik, Murat Baş

**Affiliations:** 1 Department of Nutrition and Dietetics, Institute of Health Sciences, Acibadem Mehmet Ali Aydinlar University, Istanbul 34752, Turkey; 2 Department of Endocrinology and Metabolism, Dokuz Eylül University Hospital, İzmir 35410, Turkey; 3 Department of Nutrition and Dietetics, Faculty of Health Sciences, Acibadem Mehmet Ali Aydinlar University, Istanbul 34752, Turkey; gizem.kose@acibadem.edu.tr (G.K.); esen.karaca@acibadem.edu.tr (K.E.K.Ç.); murat.bas@acibadem.edu.tr (M.B.); 4 Multidisciplinary Experimental Animal Laboratory, Faculty of Medicine, Dokuz Eylül University, İzmir 35410, Turkey; asli.celik@deu.edu.tr

**Keywords:** carb counting, type 1 diabetes, diet, medical nutrition therapy

## Abstract

Diabetes is a metabolic disease characterized by hyperglycemia due to impaired insulin secretion, activity, or both. Carbohydrate counting, known for optimal metabolic control, plays in the therapeutic strategy in diabetes. In the last decade, an increasing amount of research has been conducted on carbohydrate counting, and the literature on this topic has been published in academic journals. This bibliometric analysis aimed to comprehensively review and analyze publications from this period, shedding light on trends, developments, and key contributors. The Expanded Science Citation Index published by the Institute for Scientific Information Web of Science, which covers English-language articles published from 1993 to 2024, was used. We selected “carbohydrate counting”, “carbohydrate count”, “carbohydrate counts”, “carbohydrate counts”, and similar words as “TOPIC” to search for related articles. All basic information about each article were collected, including authors, countries, citations, and keywords. The findings emphasized the need for continued research in this area and to learn more about studies showing the relationship between carbohydrate counting and the pathophysiology of diabetes, treatment, complications, and technologies. This analysis summarizes the general trends and key findings of research on carbohydrate counting over the past years and provides guidance for future research.

## 1. Introduction

Diabetes is a metabolic disease characterized by hyperglycemia due to impaired insulin secretion, activity, or both [1]. The International Diabetes Federation has reported that the number of patients with diabetes (types 1 and 2) increased from 285 million in 2009 to 425 million in 2017 and has estimated that this number will reach 628.6 million in 2045 [2,3].

The following are the three main types of diabetes: type 1 diabetes mellitus (T1DM), type 2 diabetes mellitus (T2DM), and gestational diabetes [3]. T2DM is the most common type of DM that is characterized by hyperglycemia, insulin resistance, and relative insulin deficiency. In 1936, Sir Himsworth of England mentioned insulin sensitivity for the first time.

Thus, the distinction between types 1 and 2 diabetes began to be made [4]. T2DM is the most common type of diabetes worldwide and is a significant chronic metabolic disease as it causes acute metabolic complications, including diabetic ketoacidosis, hypoglycemia-induced coma, long-term macrovascular complications (coronary heart disease, peripheral vascular disease, and cerebrovascular disease), and microvascular complications (neuropathy, nephropathy, and retinopathy) [5,6]. According to [7], patients with T2DM usually do not require exogenous insulin; however, it may be necessary when blood glucose levels are not well controlled with oral hypoglycemic drugs alone or diet.

The relationship between diabetes and diet has been proven, and diet is as essential as medical interventions in diabetes treatment [8]. Diet plays a significant role in the therapeutic strategy for achieving glycemic control and preventing micro- and macrovascular complications in patients with diabetes [9]. 

T1DM is a chronic disease characterized by insulin deficiency that develops because of damage to the beta cells of the pancreas, thereby causing carbohydrate, protein, and fat metabolism disorders. In its treatment, insulin therapy, nutrition, and exercise are inseparable. Nutritional therapy in T1DM is based on healthy eating principles and covers the whole family. Nutritional therapy aims to achieve optimal glycemic control, maintenance of normal growth and development, promotion of lifelong healthy eating habits, and prevention of diabetes-related complications. The individualization of treatment and the provision of a nutrition plan that is appropriate to the social, psychological, cultural, and economic needs of the individual with diabetes are keys to the success of nutrition therapy. In T1DM, medical nutrition therapy is significant for complication prevention and metabolic control. Along with basic nutritional recommendations, the carbohydrate counting method is the gold standard for optimal metabolic control [10]. Carbohydrate counting is an eating plan for patients with T1DM treated with bolus insulin via multiple daily injections or continuous subcutaneous insulin infusions [6]. Carbohydrate counting focuses on carbohydrate as the primary nutrient affecting the postprandial glycemic response and assumes a linear correlation between the amount of carbohydrates consumed, mealtime, and insulin dose [7,8]. Bolus insulin is calculated from the total amount of carbohydrate consumed at each meal and the insulin-carbohydrate ratio. Studies show that carbohydrate counting may have positive effects on metabolic control and decrease glycated hemoglobin (HbA1c) concentration [9,10,11]. It is carbohydrates in meals that mainly affect the postprandial blood glucose level and determine the insulin requirement. Postprandial glycemic response and insulin requirement depend on the amount of carbohydrate in the meal rather than the type of carbohydrate consumed [5,12]. Therefore, carbohydrate counting improves glycemic control and modifies the effect of carbohydrate intake on blood glucose, thus optimizing food choices and setting nutritional goals [13]. In this respect, the carbohydrate counting method plays a significant role in achieving glycemic control goals. A diabetes dietitian, preferably a member of the diabetes team and with experience in diabetes, is recommended to start nutrition and carbohydrate counting training as soon as possible following diagnosis [10].

In the light of all this information, the carbohydrate counting method not only contributes to the improvement of glucose values and adjustment of insulin doses in individuals with diabetes but also offers these individuals a more comfortable quality of life and the opportunity to be protected from acute and chronic complications related to diabetes by offering more flexible nutritional options. Thus, it will also reduce the social burden by contributing to the Ministry of Health’s policies to combat diabetes.

In the last decade, an increasing amount of research has been conducted on carbohydrate counting, and the literature on this topic has been published in academic journals [11]. 

In this study, a comprehensive quantitative and visual analysis of the literature on T1DM and carbohydrate counting was performed through bibliometric analysis with the help of VOSviewer. This bibliometric analysis aimed to comprehensively review and analyze publications from this period, shedding light on trends, developments, and key contributors in the field. In addition, for clinicians and academicians, the results of this study will not only provide information about the important points of research in this field but also provide important research directions. 

## 2. Materials and Methods

### 2.1. Sourcing and Search Strategy

The Expanded Science Citation Index published by the Institute for Scientific Information Web of Science, which covers English-language articles published in the last 32 years (1993–2024), was used. We selected “carbohydrate counting”, “carbohydrate count”, “carbohydrate counts”, “carbohydrate counts”, and similar words (unabbreviated and singular/plural, abbreviated and singular/plural, abbreviated and singular/plural) as “TOPIC” to search for related articles (Table 1) [12,13]. We collected all basic information about each article, including authors, countries, institutions, citations, keywords, and references.

### 2.2. Inclusion Criteria

Articles should meet the following standards: publication years up to 2024, published in English, and words included in Table 1 were restricted to “OR” as topic (searches title, abstract, and author keywords).

### 2.3. Data Retrieval

Microsoft Office Excel 2019 (version 16.30) was used for the organization and analysis of data from the WOS. While exporting raw data, certain headings were selected in the WOS filtering, and information was collected. These information included publication year, authors, article title, source title, language, document type, author keywords, keywords plus, abstract, addresses, links, reprint addresses, email addresses, researcher IDs, ORCIDS, funding organizations, funding name, preferred funding text, number of references cited, times cited (WOS core), times cited (all databases), publisher, publisher city, publisher address, publication date, publication year, volume, issue, supplement, special issue, start page, end page, article number, DOI, DOI link, page number, WOS categories, research areas, open access definitions, high citation status, hot article status, export date, and WOS ID. Web of Science records are presented in the appendix. Editing of exported data and correction of errors (year of publication) were manually performed.

### 2.4. Visualization

VOSviewer, a powerful tool for bibliometric analysis, was instrumental in processing and visualizing the data received. The software allowed the construction of common citation graphs that provide visual representations of the most influential authors, nations, and organizations contributing to the field of “carbohydrate counting.” A focused analysis was performed on the most prominent keywords that exhibited significant citation bursts, shedding light on emerging trends and research directions. The top 10 most cited papers were identified, providing insights into seminal work that has significantly impacted the field. The numbers of publications, citations, and H-indexes were aggregated and presented in a yearly bar chart, providing a visual depiction of the temporal evolution of research activity. To construct a geographical representation of the regions contributing to the literature on “carbohydrate counting”, VOSviewer was used. This visualization offered insights into the global distribution of research efforts.

### 2.5. Analysis Method 

The development of scientifically based knowledge networks including countries/regions where articles were published, journals, and keywords was achieved using VOSviewer 1.6.20. Visualization of figures, graphs, and tables was performed using Microsoft Excel Office 2019. Data were exported on 22 April 2024. 

The Pearson correlation test in SPSS version 29 was used for analyzing the relationship between the total number of citations and the number of articles, average citations per article, and H-index.

### 2.6. Limitations of the Study

Only articles published in the English language from the WOS were quantitatively shown. It was limited to studies on carbohydrate counting.

## 3. Results

Without any filtering, 530 articles from 1993 to 2024 (data collection, 22 April 2024) for the keywords listed in Table 1 were identified. Overall, 514 articles met the inclusion criteria (data are included in Supplementary Materials). These included articles were cited 6006 times. Article counts, citation rates, H-index, and top 10 (T10) lists for different topics are presented. 

### 3.1. Distribution of the Total Number of Articles, Citations, and H-Index by Years

The total number of 514 articles by year varies between 0 and 57. Most articles were published in 2021 (10.31%). No articles were published in 1996 and 2002. The number of articles did not show a steady increase over the years. The mean was 17, and the median was 12.

Overall, 6078 citations ranging from 0 to 595 were noted. The mean was 190, whereas the median was 93. Articles in 2014 had the highest number of citations with 595. Articles were not cited in 1996, 2002, 2005, and 2024. Between 1993 and 2024, the average citations per article was at least 0 and at most 92, with a mean of 16.4 and a median of 12.9.

The H-index was 40 for the included years. The maximum value was 13 in 2018, and the minimum value was 0 in 1996, 2002, 2005, and 2024. The mean H-index value of the data was 4.8, and the median was 3.0.

**Figure 1:**
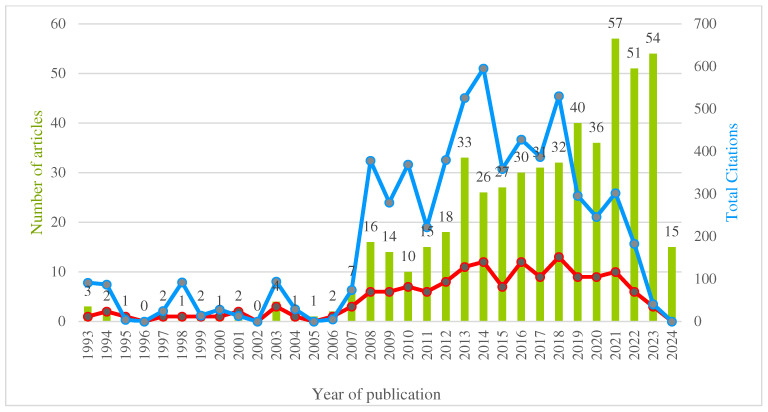
Time intervals of cited articles. Green bars represent the number of publications per year, the blue line represents the number of citations per article per year, and the red line represents the H-index by year. The distribution of articles and total number of citations by year is shown in the figure.

Total number of citations was positively correlated with H-indexes and total number of articles over the last 32 years (Pearson correlation coefficient = 0.947 and 0.567, respectively) (Figure 1). No correlation was observed between total number of citations and average number of citations per item (Pearson correlation coefficient = 0.120).

The most cited article of all time was “Impact of fat, protein, and glycemic index on postprandial glucose control in type 1 diabetes: implications for intensive diabetes management in the continuous glucose monitoring era” by Bell, Kirstine J et al., published in *Diabetes Care* in 2015 (Publisher name: American Diabetes Association) [14].

### 3.2. Analysis of Cited Articles

The T10 list in descending order according to the total number of citations of English articles is presented in Table 2. The most cited T10 articles in the total process were published between 1993 and 2017. Diabetes Care published the most cited article (n = 243), followed by The Lancet (n = 165) and Diabetes Research and Clinical Practice (n = 155). The T10 articles had a mean citation count of 137.3 and a median of 131.

### 3.3. Author Analysis by Number of Publications

The T10 list in descending order according to the total number of publications by corresponding author is shown in Table 3. According to the data meeting the inclusion criteria, the author with the most publications was Haidar A. (n = 21), followed by Legault L. (n = 15), and Norgaard K. (n = 15). The mean number of publications by the T10 authors was 11.5, and the median was 9.

### 3.4. Co-Authorship of Authors Analysis

Co-authors were identified within the restricted articles in accordance with the inclusion criteria. For this purpose, the authors were determined to have at least two publications and at least one citation. Accordingly, from a total of 2452 authors, 389 data points were obtained.

**Figure 2:**
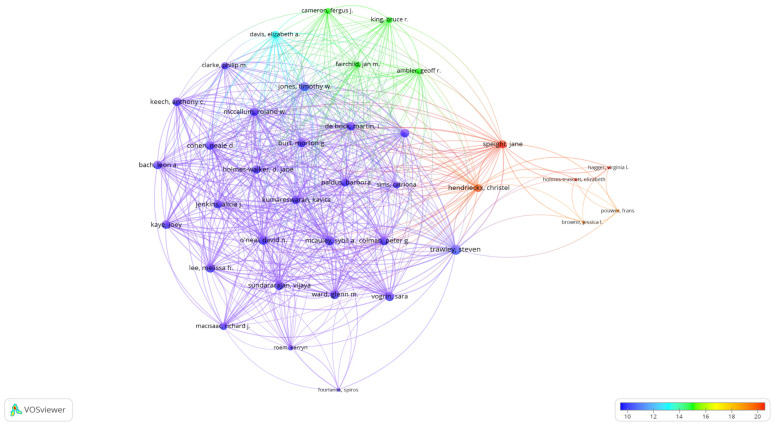
Visualization of the co-authorship of authors. Scale, 1.0; weights, total link strength; scores, average citations.

The author with the highest number of co-authorships was Trawley Steven with 97 co-authorships and seven documents with 73 total citations. Haidar Ahmad was the most cited author with 95 total link strength and 157 total citations in 16 articles. Mcauley Sybil A. followed as the most cited author with 94 total link strength and 47 citations in six articles (Figure 2).

### 3.5. Citation Analysis of Authors (Citations of Authors)

**Figure 3:**
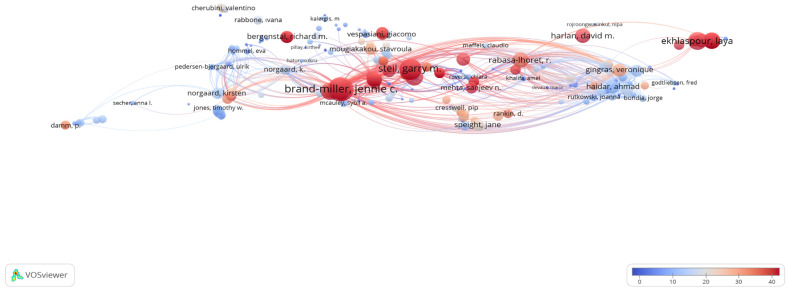
Visualization of the citations of authors. Scale, 1.0; weights, citations; scores, average citations.

In accordance with the inclusion criteria, citation analysis of the authors was performed within the limited articles. For this purpose, the authors were determined to have at least two publications and at least two citations. Accordingly, 381 data points were obtained from 2452 authors. The author with the highest number of citations was Brand-Miller Jennie C., with 514 citations in eight articles. This was followed by Bell Kirstine J. with 389 citations in four articles and Steail Garry M. with 375 citations in two articles (Figure 3).

### 3.6. Publishers

Articles that met the inclusion criteria were published by several publishers. The largest T10 publisher was Elsevier (n = 72), followed by Wiley (n = 68), and the American Diabetes Association (n = 59) (Table 4). The T10 publishers published an average of 38.3 articles with a median of 38.5.

The T10 journals that published the most number of articles related to the TOPICs that met the inclusion criteria are presented in Table 5. Diabetes Technology Therapeutics (n = 58) was the journal with the highest number of publications during the period specified in the inclusion criteria, followed by Diabetes (n = 43) and Diabetic Medicine (n = 33). The average number of publications by the T10 journals in this field was 22.4, and the median was 15.5.

### 3.7. Citations of Journals

**Figure 4:**
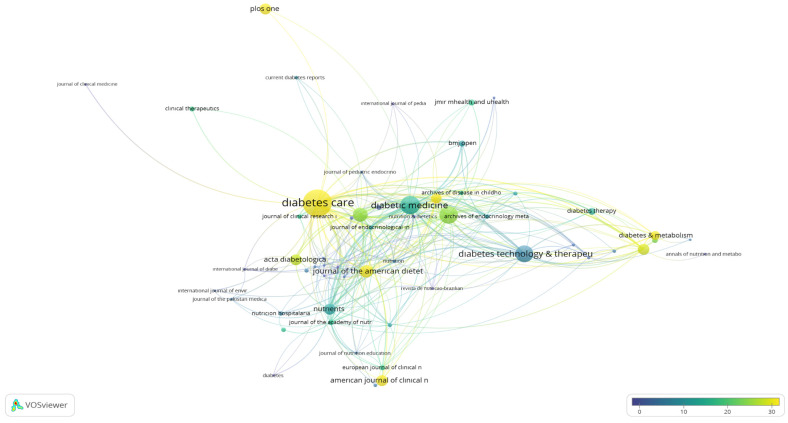
Visualization of the citations of journals. Scale, 1.0; weights, citations; scores, average citations.

Journals cited in the restricted documents in accordance with the inclusion criteria were identified. Accordingly, 194 journals had citations. The minimum number of articles and citations of a journal was set to two and one, respectively, and were subsequently analyzed; 59 data points were obtained. The cited journals are shown in Figure 4. The most cited journal was Diabetes Care (1037 citations; 16 documents). This was followed by Diabetic Medicine (456 citations; 33 documents), Diabetes Research and Clinical Practice (429 citations; 18 documents), Diabetes Technology & Therapeutics (402 citations; 58 documents), and Pediatric Diabetes (264 citations; 11 documents), in descending order.

### 3.8. Broadcasting Countries

The T10 countries that have contributed the most to the field according to the articles that met the inclusion criteria are shown in Table 6. The largest contribution to the field over the 32-year period came from the USA, followed by England and Australia (record count, 117, 58, and 40, respectively).

### 3.9. Cited Countries

**Figure 5:**
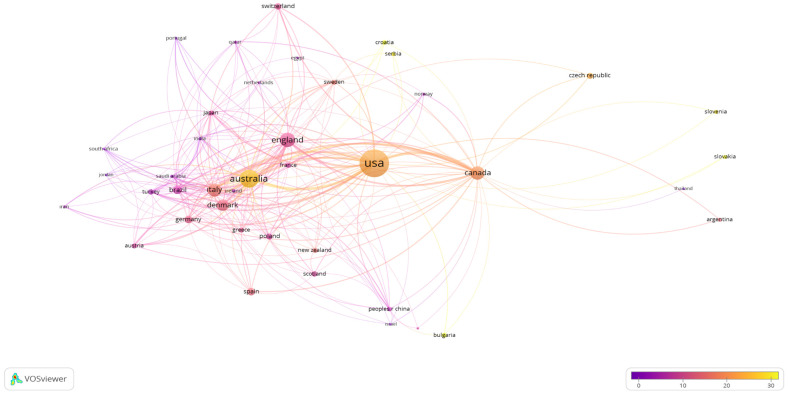
Visualisation of cited countries. Scale, 1.0; weights, citations; scores, average citations.

Countries that were cited in the restricted articles in accordance with the inclusion criteria were identified. Accordingly, 100 countries were identified as having citations. The minimum number of documents and citations of a country was set to two and two, respectively; 42 countries were included. The 42 cited countries are depicted in Figure 5. The USA was the most cited country (2371 citations; 113 articles), followed by Australia (982 citations; 40 articles) and England (636 citations; 58 articles), in descending order.

### 3.10. Research Area Analysis

The T10 research areas with the most number of studies in the TOPICs indicated in Table 7 are presented in descending order in Table 6. In the T10 research areas, endocrinology metabolism (60.89%) was the most popular research area, followed by studies on nutrition dietetics (14.20%) and pediatrics (7.39%). The average number of publications in the T10 listed research areas was 54.0 with a median of 16.0.

### 3.11. Analysis of Keywords

**Figure 6:**
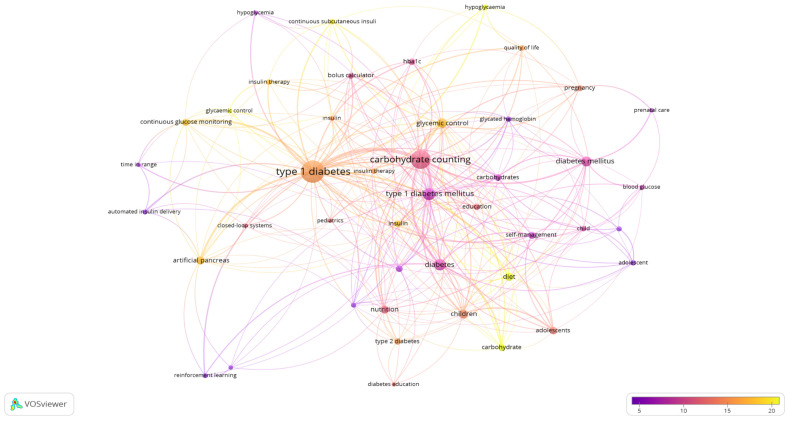
Visualization of the occurrences of keywords. Scale, 1.0; weights, occurrences; scores, average citations.

The total number of keywords in the articles that met the inclusion criteria was 720. The minimum number of occurrences of a keyword was five, and the number was 44 according to this limitation (Figure 6). The keywords with occurrences in descending order were type 1 diabetes (n = 120), carbohydrate counting (n = 84), type 1 DM (n = 35), diabetes (n = 33), DM (n = 24), and glycemic control (n = 21).

### 3.12. Document Types

**Figure 7:**
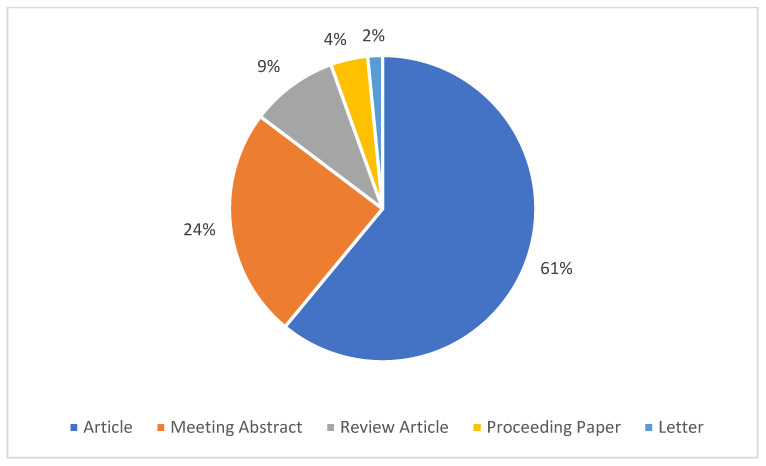
Percentage scatter plot of the top five document types.

Documents with different characteristics that met the inclusion criteria were identified. In descending order, the document types were articles (n = 311), meeting abstracts (n = 124), review articles (n = 47), proceeding papers (n = 20), letters (n = 8), book chapters (n = 7), editorial materials (n = 7), early access (n = 4), retracted publication (n = 1), and retraction (n = 1). The scatter plot of the documents that met the inclusion criteria is depicted in Figure 7.

## 4. Discussion

This study provides a comprehensive review of carbohydrate counting-related research on diabetes and nutrition over the last 32 years and sheds light on trends, developments, and key contributors in the field through bibliometric analyses. The findings show that a wide range of research exists on carbohydrate counting, and a significant interest is observed in this field.

Over the last 32 years, research on carbohydrate counting has shown no particular trend or increase. The average number of citations and H-index values has remained relatively stable. The number of articles and citations has varied over the years. A positive correlation was noted between total number of citations, H-indexes, and total number of articles over the 32-year period. No correlation was observed between total number of citations and average citations per item, indicating that each article has a different impact and citation potential.

These stable trends highlight that the dynamics of carbohydrate counting research are less variable compared to other research fields. For example, research on dietary interventions in diabetes has shown significant increases in both publication volume and citation rates. This growth reflects a rising recognition of the importance of diet in diabetes management and the broader application of these studies. Similarly, in this study, diabetes research has experienced significant shifts in focus and increases in both publication and citation metrics. This expansion includes various topics such as new treatment modalities, innovative monitoring technologies, and comprehensive management strategies.

In this study, all studies examining the effect of dietary fat, protein, and glycemic index have shown that these dietary factors alter postprandial glycemia. In late postprandial hyperglycemia, the effect of dietary fat is relatively large, whereas some studies have also shown that glucose concentrations decrease in the first 2–3 h owing to a possible delay in the gastric emptying rate. Carbohydrate counting provides a better understanding of the insulin bolus dose and delivery pattern required for high-fat and/or high-protein meals. Owing to methodological differences and limitations in experimental design, study findings were inconsistent regarding the optimal bolus delivery pattern; however, studies have shown that high-fat/protein meals require more insulin than low-fat/protein meals with the same carbohydrate content [14].

While carbohydrate counting remains a valuable tool for understanding and managing postprandial glycemia, ongoing research is necessary to address the methodological challenges and refine insulin delivery practices. Future studies should focus on standardizing methodologies, exploring the interactions between dietary components and insulin dynamics, and developing more personalized approaches to insulin therapy. By doing so, researchers can enhance the accuracy and effectiveness of carbohydrate counting and improve diabetes management outcomes.

Although it has been reported that superior glycemic regulation can be achieved without the need for carbohydrate counting in bihormonal bionic pancreas systems compared with treatment using insulin pump systems that cannot be used without carbohydrate counting training, larger and longer studies are needed to determine the long-term benefits and risks of automated glycemia management using bihormonal bionic pancreas systems [15]. While bihormonal bionic pancreas systems represent a promising advancement in diabetes management by potentially reducing reliance on carbohydrate counting, more comprehensive and long-term studies are essential. Such research will provide a clearer picture of their effectiveness, safety, and practical implications, ultimately guiding their implementation and integration into standard diabetes care practices.

Analyzing the most cited articles showed that a wide range of studies have been conducted on carbohydrate counting and that the diversity and depth of research in this field are observed. Among these studies, significant findings focusing on the role of carbohydrate counting in the type, course, and especially the treatment of diabetes and its contribution to diabetes technology are noted. The most cited article was Bell et al.’s 2015 Diabetes Care article entitled “Impact of Fat, Protein, and Glycemic Index on Postprandial Glucose Control in T1DM: Implications for Intensive Diabetes Management in the Continuous Glucose Monitoring Era” [14]. This article plays a significant role in the field of diabetes, especially regarding blood glucose regulation in terms of carbohydrate counting, examining the effect of dietary protein and fat on postprandial blood glucose levels, and clarifying the issue. In this study, the breadth and impact of the research on carbohydrate counting, as demonstrated by highly cited studies such as Bell et al.’s, underscore the importance of ongoing investigation in this area. These studies not only contribute to our understanding of carbohydrate counting but also inform practical strategies for diabetes management. Continued research and the application of these insights will be crucial for advancing diabetes care and enhancing the efficacy of current management practices.

The analysis of co-authorship between authors shows that research in this field is frequently conducted with contributions from more than one researcher. The most co-authored author was Brawley Steven. The most cited author was Haidar Ahmad.

Moreover, among the articles that met the inclusion criteria, Brand-Miller Jennie C. was the author with the highest number of citations, with 514 citations in eight articles. This was followed by Bell Kirstine J. with 389 citations in 4 articles [14,15,16,17,18]. These results suggest that carbohydrate counting is a meal planning method that can be preferred in the medical nutrition therapy of diabetes. Of note, this method should not focus only on the amount of carbohydrate in the meal, and the protein–fat content of the meal should not be ignored when calculating the optimal insulin dose. Studies are needed to evaluate several other factors that are related to blood glucose to evaluate the above-mentioned factors [19,20,21,22,23]. While carbohydrate counting remains a fundamental aspect of diabetes management, it is essential to integrate it with a broader understanding of how protein and fat content impact glucose control. Continued research into these factors will be critical for refining diabetes care strategies and improving the effectiveness of meal planning approaches.

Research on carbohydrate counting reaches a wide audience through various publishers. In this context, Elsevier published the most number of articles [24]. This is followed by Wiley and American Diabetes Association. This finding shows that research on carbohydrate counting is published through various publishers and that this topic is widespread in the scientific literature [25,26]. These publishers play a significant role in publishing and disseminating the work of researchers to a wide audience.

The fact that Diabetes Technology Therapeutics was the most widely published and cited journal in this field can be attributed to the fact that the journal is a multidisciplinary platform and reaches a wide readership owing to its open access policy. Diabetes Technology Therapeutics provides practical and comprehensive information on the latest technologies and treatments in the field, and research on carbohydrate counting is also likely to be published in this journal. The high rankings of Diabetes and Diabetic Medicine, followed by Diabetes and Diabetic Medicine, respectively, may be related to the fact that these journals specialize in diabetes and diabetes treatments [27]. The carbohydrate counting method may attract significant interest and may be published in these journals as it is an accepted method within medical nutrition therapy among the treatment options of diabetes and a method that should be learned as a priority in diabetes technologies. Furthermore, the fact that pediatric group journals, which are among the cited journals, are essential in terms of the fact that diabetes starts at an early age, even in infancy/childhood, and carbohydrate counting education is highly significant with respect to fostering positive results in the treatment of these age groups highlights its importance in diabetes research. In this study, the prominence of carbohydrate counting in diabetes research and its publication in key journals demonstrate its significant role in the field. Continued research and dissemination in both general and pediatric diabetes contexts are crucial for advancing knowledge, refining treatment strategies, and enhancing patient care across all age groups.

A review of countries’ research on carbohydrate counting revealed that the USA (117) is the leading contributor in this field with a significant number of publications and citations. This finding points to a role for the USA’s emphasis on carbohydrate counting in diabetes treatment [28,29,30]. England and Australia follow the USA with 58 and 40 publications, respectively [31,32,33,34]. Italy and Denmark follow in the ranking of contributing countries. Brazil and Canada come next, and their citation numbers are close to each other. Moreover, Turkey ranks in the top ten countries, showing its interest and importance in this field. These findings and the fact that there are several different countries working in this field underline the significance of the carbohydrate counting method in the treatment of diabetes, the incidence of which is rapidly increasing globally [35,36,37,38,39].

Analyzing carbohydrate counting-related studies reveals that there are concentrated studies and key topics in various scientific research areas. Analyzing the main focal points of the studies in this field shows that endocrinology metabolism (60.89%) is the most popular area of research in this field. This area includes strategies for improving treatment options for diabetes [40]. Studies on nutrition dietetics (14.20%) also focused on investigating medical nutrition treatment methods for diabetes, especially carbohydrate counting [41]. Studies on pediatrics (7.39%) focused on examining the metabolic effects of treatment options for childhood diabetes [42]. General internal medicine (7.2%), engineering (3.31%), medical informatics (2.92%), public environmental occupational health (2.92%), automation control systems (2.14%), computer science (2.14%), and health care sciences services (1.9%) are other relevant research areas [43,44,45,46,47,48,49]. Keywords are also related to these fields and reflect the main topics of focus in research on carbohydrate counting. Type 1 diabetes, carbohydrate counting, type 1 diabetes mellitus, type 1 diabetes mellitus, diabetes, diabetes mellitus, and glycemic control reflect the main topics that drive the work of researchers [50,51,52].

It shows that research in this field is based on a broad multidisciplinary approach and is concentrated in various research areas. Researchers have employed pharmacology, biochemistry, endocrinology, public health, computer technology, and other scientific disciplines for understanding and utilizing carbohydrate counting in different areas of diabetes treatment. By leveraging insights from diverse fields, researchers can develop more effective strategies for carbohydrate counting, ultimately improving diabetes care and patient outcomes. Continued collaboration across these disciplines will be essential for advancing our understanding and application of carbohydrate counting in managing diabetes.

## 5. Conclusions

Recent studies over the past few years have shown that high carbohydrate intake from processed and ultra-processed foods increases the risk of all types of diabetes, especially T2DM. Lifestyle and dietary behavior changes, including nutritional therapy and an appropriate exercise plan, are known to improve blood glucose regulation.

Carbohydrate counting is a meal planning practice for diabetic patients, aiming to manage blood glucose levels by tracking the amount of carbohydrates in grams consumed at meals. While it typically uses the total carbohydrate amount to determine prandial insulin needs, the type of carbohydrate can also be a factor affecting insulin requirements. Through carbohydrate counting, diabetic individuals can clearly see the negative effects of high carbohydrate intake from processed and ultra-processed foods on their blood glucose levels. The importance of physical activity and carbohydrate counting in the prevention, treatment, and management of diabetes is increasingly being highlighted.

The interest in carbohydrate counts in the last decades has been relatively steady. This study assesses the state of research on carbohydrate counting in the field of medical nutrition therapy, which is a significant pillar of diabetes treatment and provides guidance for future research. The findings emphasize the need for continued research in this area and to learn more about studies showing the relationship between carbohydrate counting and the pathophysiology of diabetes, treatment strategies, complications, and diabetes technologies.

This analysis summarizes the general trends and key findings of research on carbohydrate counting over the past and provides guidance for future research.

## Figures and Tables

**Table 1:** TOPIC list used in WOS filtering.

Unabbreviated	Abbreviated
“carbohydrate counting”	“carb counting”
“carbohydrate count”	“carb count”
“carbohydrate counts”	“carb counts”

**Table 2:** The most cited T10 articles.

Rank	Citation	Citations (Average per Year)	Authors	Title	Journal	Year	DOI
1	243	24.3	Bell, Kirstine J.; Smart, Carmel E.; Steil, Garry M.; Brand-Miller, Jennie C.; King, Bruce; Wolpert, Howard A.	Impact of Fat, Protein, and Glycemic Index on Postprandial Glucose Control in Type 1 Diabetes: Implications for Intensive Diabetes Management in the Continuous Glucose Monitoring Era [14]	Diabetes Care	2015	10.2337/dc15-0100
2	165	20.63	El-Khatib, Firas H.; Balliro, Courtney; Hillard, Mallory A.; Magyar, Kendra L.; Ekhlaspour, Laya; Sinha, Manasi; Mondesir, Debbie; Esmaeili, Aryan; Hartigan, Celia; Thompson, Michael J.; Malkani, Samir; Lock, J. Paul; Harlan, David M.; Clinton, Paula; Frank, Eliana; Wilson, Darrell M.; DeSalvo, Daniel; Norlander, Lisa; Ly, Trang; Buckingham, Bruce A.; Diner, Jamie; Dezube, Milana; Young, Laura A.; Goley, April; Kirkman, M. Sue; Buse, John B.; Zheng, Hui; Selagamsetty, Rajendranath R.; Damiano, Edward R.; Russell, Steven J.	Home Use of a Bihormonal Bionic Pancreas versus Insulin Pump Therapy in adults with Type 1 Diabetes: A Multicentre Randomised Crossover Trial [15]	The Lancet	2017	10.1016/S0140-6736(16)32567-3
3	155	12.92	Brazeau, A. S.; Mircescu, H.; Desjardins, K.; Leroux, C.; Strychar, I.; Ekoe, J. M.; Rabasa-Lhoret, R.	Carbohydrate Counting Accuracy and Blood Glucose Variability in Adults with Type 1 Diabetes [16]	Diabetes Research and Clinical Practice	2013	10.1016/j.diabres.2012.10.024
4	132	12	Saslow, Laura R.; Kim, Sarah; Daubenmier, Jennifer J.; Moskowitz, Judith T.; Phinney, Stephen D.; Goldman, Veronica; Murphy, Elizabeth J.; Cox, Rachel M.; Moran, Patricia; Hecht, Fredrick M.	A Randomized Pilot Trial of a Moderate Carbohydrate Diet Compared to a Very Low Carbohydrate Diet in Overweight or Obese Individuals with Type 2 Diabetes Mellitus or Prediabetes [17]	PLOS One	2014	10.1371/journal.pone.0091027
5	132	11	Wolpert, Howard A.; Atakov-Castillo, Astrid; Smith, Stephanie A.; Steil, Garry M.	Dietary Fat Acutely Increases Glucose Concentrations and Insulin Requirements in Patients with Type 1 Diabetes Implications for Carbohydrate-Based Bolus Dose Calculation and Intensive Diabetes Management [18]	Diabetes Care	2013	10.2337/dc12-0092
6	130	11.82	Bell, Kirstine J.; Barclay, Alan W.; Petocz, Peter; Colagiuri, Stephen; Brand-Miller, Jennie C.	Efficacy of Carbohydrate Counting in Type 1 Diabetes: A Systematic Review and Meta-analysis [19]	The Lancet Diabetes & Endocrinology	2014	10.1016/S2213-8587(13)70144-X
7	121	7.12	Bergenstal, Richard M.; Johnson, Mary; Powers, Margaret A.; Wynne, Alan; Vlajnic, Aleksandra; Hollander, Priscilla; Rendell, Marc	Adjust to Target in Type 2 Diabetes: Comparison of a Simple Algorithm with Carbohydrate Counting for Adjustment of Mealtime Insulin Glulisine [20]	Diabetes Care	2008	10.2337/dc07-2137
8	113	7.53	Rossi, Maria C. E.; Nicolucci, Antonio; Di Bartolo, Paolo; Bruttomesso, Daniela; Girelli, Angela; Ampudia, Francisco J.; Kerr, David; Ceriello, Antonio; De La Questa Mayor, Carmen; Pellegrini, Fabio; Horwitz, David; Vespasiani, Giacomo	Diabetes Interactive Diary: A New Telemedicine System Enabling Flexible Diet and Insulin Therapy While Improving Quality of Life an Open-Label International Multicenter Randomized Study [21]	Diabetes Care	2010	10.2337/dc09-1327
9	92	3.41	Gillespie, SJ; Kulkarni, KD; Daly, AE	Using Carbohydrate Counting in Diabetes Clinical Practice [22]	Journal of the American Dietetic Association	1998	10.1016/S0002-8223(98)00206-5
10	90	2.81	Anderson, EJ; Richardson, M; Castle, G; Cercone, S; Delahanty, L; Lyon, R; Mueller, D; Snetselaar, L	Nutrition Interventions for Intensive Therapy In The Diabetes Control And Complications Trial [23]	Journal of the American Dietetic Association	1993	10.1016/0002-8223(93)91750-K

**Table 3:** T10 authors with the most number of publications.

Rank	Authors	Record Count	% of 0.514
1	Haidar A	21	4.086
2	Legault L	15	2.918
3	Norgaard K	15	2.918
4	Rabasa-lhoret R	13	2.529
5	Brand-miller JC	9	1.751
6	El Fathi A	9	1.751
7	Yale JF	9	1.751
8	Hommel E	8	1.556
9	Mathiesen ER	8	1.556
10	Schmidt S	8	1.556

**Table 4:** List of T10 publishers with the most article publications.

Rank	Publishers	Record Count	% of 0.514
1	Elsevier	72	14.008
2	Wiley	68	13.230
3	Amer Diabetes Assoc	59	11.479
4	Springer Nature	59	11.479
5	Mary Ann Liebert, Inc	54	10.506
6	Mdpi	23	4.475
7	Karger	21	4.086
8	Sage	11	2.140
9	Bmj Publishing Group	9	1.751
10	IEEE	7	1.362

**Table 5:** T10 journals with the most number of publications.

Rank	Publication Titles	Record Count	% of 0.514
1	Diabetes Technology Therapeutics	58	11.28
2	Diabetes	43	8.37
3	Diabetic Medicine	33	6.42
4	Diabetes Research And Clinical Practice	18	3.50
5	Diabetes Care	16	3.11
6	Diabetologia	15	2.92
7	Nutrients	15	2.92
8	Pediatric Diabetes	11	2.14
9	Practical Diabetes	8	1.56
10	Annals Of Nutrition And Metabolism	7	1.36

**Table 6:** T10 contributing countries.

Rank	Countries/Regions	Record Count	% of 0.514
1	USA	117	22.763
2	England	58	11.284
3	Australia	40	7.782
4	Italy	34	6.615
5	Denmark	30	5.837
6	Brazil	29	5.642
7	Canada	29	5.642
8	Turkey	17	3.307
9	Germany	14	2.724
10	India	13	2.529

**Table 7:** T10 research areas with the most number of studies.

Rank	Research Areas	Record Count	% of 0.514
1	Endocrinology Metabolism	313	60.89
2	Nutrition Dietetics	73	14.20
3	Pediatrics	38	7.39
4	General Internal Medicine	37	7.20
5	Engineering	17	3.31
6	Medical Informatics	15	2.92
7	Public Environmental Occupational Health	15	2.92
8	Automation Control Systems	11	2.14
9	Computer Science	11	2.14
10	Health Care Sciences Services	10	1.95

## Data Availability

Data is contained within the article and Supplementary Materials.

## Supplementary Materials

The following supporting information can be downloaded at: https://www.mdpi.com/article/10.3390/nu16193249/s1. The content of 514 articles indicating the inclusion criteria is present in the supplement. It includes information such as Type of Publication, Author Names and Surnames, Article Title, Document Type, Author Keywords, Affiliated Organisations, Funding, Number of References Cited and Time Cited etc.
